# Genome Sequence of the Arsenic-Resistant *Haladaptatus* sp. Strain R4 Isolated from Ramnagar, West Bengal, India

**DOI:** 10.1128/genomeA.01017-16

**Published:** 2016-09-22

**Authors:** Urmimala Sen, Trinetra Mukherjee, Sucharita Bose, Chayan Roy, Moidu Jameela Rameez, Wriddhiman Ghosh, Subhra Kanti Mukhopadhyay

**Affiliations:** a Department of Microbiology, University of Burdwan, Burdwan, West Bengal, India; b Department of Microbiology, Bose Institute, Centenary Campus, Kolkata, West Bengal, India

## Abstract

Here, we present the draft genome of *Haladaptatus* sp. strain R4, a halophilic archaea that produces an orange-pink pigment and is capable of growing in a wide salinity range. The genome assembly shows genes for arsenic resistance, siderophore production, trehalose and glycine betaine biosynthesis, uptake and transporters of sodium, potassium, and chloride ions.

## GENOME ANNOUNCEMENT

Among the 40 genera under the family *Halobacteriaceae*, *Haladaptatus* is special because of its wide salinity range (1). *Haladaptatus* sp. strain R4 was isolated from a solar evaporation pond in Ramnagar (21°66′97″ N, 87°54′21″ E) in Midnapur district, West Bengal, India, and grown on halophile medium modified from Oren et al. (2). The 16S rDNA sequence of strain R4 has 98% identity with *Haladaptatus paucihalophilus* strain DX253. The coccoid cells are orange-pink pigmented and capable of growth up to 5M NaCl. The cells do not get disrupted after prolonged incubation in distilled water.

Genomic DNA was obtained by a chloroform-isoamyl alcohol extraction method. The purified genome was sequenced by Ion PGM sequencer (3) using a 318 chip. SPAdes version 3.5 was employed for assembly, which resulted in 34 contigs with a length of 4.9 Mb. The longest contig size is 1,088,768 bp, and the smallest contig size is 376 bp. The average G+C content is 60.18%. The *de-novo* assembled sequence was further annotated by submitting the sequence to the NCBI’s Prokaryotic Genome Automatic Annotation Pipeline (PGAP). A total of 4,086 protein-coding genes were obtained, and four 16S rRNAs, 49 tRNAs, two ncRNAs, and two CRISPR arrays were present in the genome. Functional annotation by the Rapid Annotations using Subsystems Technology (RAST) server (4) showed a relatively high number of coding sequences associated with carbohydrate, protein, DNA, RNA and miscellaneous metabolism in strain R4. The Pathway Annotation Tool of the Pathosystems Resource Integration Center (PATRIC) (5) and manual annotation revealed the presence of whole or almost whole pathways such as glycolysis, Kreb’s cycle, gluconeogenesis, fatty acid metabolism, halocyanin-based electron transport chain, oxidative phosphorylation, nucleotide metabolism, amino acid metabolism, amino-acyl t-RNA biosynthesis, glycol-sphingolipid, and folate biosynthesis.

It could be interpreted from the genome assembly data that both compatible solutes and salts work together to maintain the wide osmotic range. Among compatible solutes, the OtsAB pathway of trehalose biosynthesis and trehalose ABC transporters are present. Genes of the glycine betaine transporter OpuD have been found (6), and the presence of a choline-mediated glycine betaine biosynthesis pathway has been suggested. Betaine aldehyde dehydrogenase, the high-affinity choline uptake protein BetT, and a number of dehydrogenase genes, as well as monooxygenase genes whose full annotation is still not possible, are present (7). A number of symporter, antiporter, ABC transporter, and channel proteins transporting sodium, potassium, and chloride were also found. Magnesium, cobalt, nickel, zinc, and selenium transporters are also present.

Arsenic resistance has been reported in the laboratory. Annotation via PGAP, RAST, and PATRIC suggests that the archaeal strain converts arsenate into arsenite using the enzyme arsenate reductase and extrudes arsenite by an arsenite-transporting ATPase. A gene of the transcriptional regulator of the ArsR family is also present in the genome (8). The heat shock proteins GroEL, Hsp20, and HtpX, as well as cold shock proteins and phage shock proteins are present. Siderophore-producing enzymes are found in the genomic assembly and have been supported by laboratory experiment. The genome of strain R4 will provide insights into its wide adaptation and stress responses.

### Accession number(s).

This whole-genome shotgun project has been deposited at DDBJ/ENA/GenBank under the accession number LWHG00000000. The version described in this paper is the first version, LWHG01000000.
